# Quantitative Assessment of First Annular Pulley and Adjacent Tissues Using High-Frequency Ultrasound

**DOI:** 10.3390/s17010107

**Published:** 2017-01-07

**Authors:** Yi-Hsun Lin, Tai-Hua Yang, Shyh-Hau Wang, Fong-Chin Su

**Affiliations:** 1 Department of Computer Science and Information Engineering & Institute of Medical Informatics, National Cheng Kung University, No. 1, University Road, Tainan City 70101, Taiwan; hsun224@gmail.com; 2 Department of Biomedical Engineering, National Cheng Kung University, No. 1, University Road, Tainan City 70101, Taiwan; dd2006tw@gmail.com (T.-H.Y.); fcsu@mail.ncku.edu.tw (F.-C.S.); 3 Medical Device Innovation Center, National Cheng Kung University, No. 1, University Road, Tainan City 70101, Taiwan

**Keywords:** pulley, attenuation coefficient, sound speed, integrated backscatter, Nakagami parameter

## Abstract

Due to a lack of appropriate image resolution, most ultrasound scanners are unable to sensitively discern the pulley tissues. To extensively investigate the properties of the A1 pulley system and the surrounding tissues for assessing trigger finger, a 30 MHz ultrasound system was implemented to perform in vitro experiments using the hypodermis, A1 pulley, and superficial digital flexor tendon (SDFT) dissected from cadavers. Ultrasound signals were acquired from both the transverse and sagittal planes of each tissue sample. The quantitative ultrasonic parameters, including sound speed, attenuation coefficient, integrated backscatter (*IB*) and Nakagami parameter (*m*), were subsequently estimated to characterize the tissue properties. The results demonstrated that the acquired ultrasound images have high resolution and are able to sufficiently differentiate the variations of tissue textures. Moreover, the attenuation slope of the hypodermis is larger than those of the A1 pulley and SDFT. The *IB* of A1 pulley is about the same as that of the hypodermis, and is very different from SDFT. The *m* parameter of the A1 pulley is also very different from those of hypodermis and SDFT. This study demonstrated that high-frequency ultrasound images in conjunction with ultrasonic parameters are capable of characterizing the A1 pulley system and surrounding tissues.

## 1. Introduction

Pulley is a crucial tissue that extends from the head of the metacarpal bones to the base of the distal phalanges at eight specific points along the tendon sheath [1]. Together with the flexor tendons to close to the phalanges, the pulley tissue plays an integral role in controlling the movements of finger flexion and extension [2]. The primary function of pulley tissues is to provide a fulcrum for transferring the force of the muscle-tendon unit into rotation and torque at the finger joints [3]. The anatomic morphology of pulley tissues generally can be classified into five annular pulleys (named A1 to A5) formed by thick arciform fibers, and three cruciate pulleys (named C1 to C3) formed by thin crisscrossing fibers [4]. The A1, A3, and A5 pulleys are located at the metacarpophalangeal, proximal interphalangeal, and distal interphalangeal joints, respectively, and those of A2 and A4 pulleys are located respectively at the proximal and middle phalanx. The locations of C1, C2, and C3 are respectively within the space between the A2 and A3 pulleys, the A3 and A4 pulleys, and the distal A4 and A5 pulleys [5]. The annular pulleys prevent the tendon from bowstringing during finger flexion, whereas the cruciate pulleys allow the annular pulleys to move close to one another [6]. Therefore, a lesion in the pulley tissues may immediately result in a reduction of digital performance, such as fixed flexion contracture of the proximal interphalangeal joint and loss of strength and decreasing finger motion function [7]. Among those finger motion complications, so-called trigger finger is a common malady causing the blockage of finger movement from flexion to extension that is primarily associated with pathological pulley tissues [8]. Specifically, histological assessment reveals that trigger finger is mainly a result of fibrocartilaginous metaplasia in the A1 pulley and superficial tendons [9]. Moreover, several factors, such as overuse injuries, diabetes mellitus, rheumatoid arthritis, gout, glycogen storage disease, and acromegaly, have been found to cause trigger finger [8].

To date, trigger finger diagnosis is generally based on first reviewing patients’ medical history and then apply physical examinations, such as the assessment of finger movement and the palpation of any swollen or painful area around the pulley region [8,10]. The diagnostic accuracy of physical examinations is largely dependent on each physician’s clinical experience, so it is essential to improve the accuracy of assessment of the degree of pulley disease for further therapeutic management. An appropriate instrument able to objectively aid in diagnosing trigger finger is very necessary. Several researchers have tried to apply ultrasonic B-mode imaging, which is non-invasive and has real-time imaging capability, to assess pulley tissue lesions. For example, 12 MHz ultrasound images have been used to directly visualize annular pulley lesions, however it was still difficult to assess the injuries of pulley structure and to discern the normal pulley [1,10,11]. The normal pulley tissues, except for the A5, C2, and C3 pulleys, could be discerned when the ultrasound frequency was increased to 17 MHz [1]. The B-mode ultrasound image of normal annular pulleys appears as hyperechogenic structures, and whereas the annular pulley of trigger finger tends to be hypoechogenic relative to those of the normal pulleys [12,13,14]. The thickness of A1 pulleys with trigger finger was measured to vary from 1.1 to 2.9 mm, with an average of 1.8 mm, which is a significantly greater value than that seen in normal fingers, which varies from 0.4 to 0.6 mm, with an average of 0.5 mm [12]. Moreover, the power Doppler imaging corresponding to A1 pulleys has been found to be show 91% hypervascularization compared to patients with trigger finger [12]. Another interesting study for estimating the tissue stiffness was conducted by measuring the strain ratio (F/P strain ratio) between the subcutaneous fat and A1 pulley tissues [15]. The F/P strain ratio of the trigger finger (4.2 ± 1.3) was found to be much stiffer than that of the adjacent normal fingers (2.4 ± 0.63). In addition to direct measurement of A1 pulley, other measurement features, including tendinosis, blurred margins, and irregular echogenic textures of the flexor tendon under A1 pulley lesion conditions have also been applied to assess trigger finger [9,12,16]. However, the resolution of 17 MHz clinical ultrasound scanners remains inadequate to sensitively discern the thickness and complexity of annular pulleys in normal fingers. It is known that the resolution and sensitivity of ultrasound images can be readily improved by increasing the ultrasound frequency. Studies applying ultrasounds with frequencies higher than 20 MHz were found able to measure such cells and fine tissues as the blood, fibroblasts, skin, eye, vasculature, and that of small animals [17,18,19,20,21,22]. Hence, it should be feasible to obtain more detailed information on pulley tissues with high-frequency ultrasound.

With its real-time imaging features, ultrasound B-mode imagery is capable of quickly examining the cross-sectional anatomical structure of interrogated tissues, yet, the quality of ultrasound images may be easily affected by the scanner settings such as system gain, time-gain compensation, dynamic range, and other procedures related to the signal and image processing scheme. Therefore, the B-mode images tend to provide more qualitative tissue information and their quality is operator-dependent. On the other hand, ultrasonic parameters, such as sound speed, frequency-dependent attenuation, and backscattering coefficients, are well known to correspond to biological tissue properties and have been applied to quantitatively characterize tissue features such as blood hematocrit, blood coagulation, skin fibrosis, degree of skin burns, and differentiation of the dermis layer [20,23,24,25,26]. The apparent integrated backscatter (*IB*) applied to assess the human Achilles tendon as a function of insonification angle was found to also be related to the fiber axis of the tendon [27]. The maximum apparent *IB* was obtained from ultrasonic signals corresponding to the insonification angle which is perpendicular to the tendon fibers, whereas the minimum apparent *IB* came from those parallel to the fibers.

In addition to ultrasonic backscattered strength, the probability density functions (PDF) of backscattering signals could be another means to characterize tissue properties due to the fact that the complexity and composition of biological tissues tends to have a random nature and which may accordingly result in different ultrasonic backscattered strength distributions. This enables the analysis of the statistical distribution of ultrasonic backscattered envelopes to be correlated to tissue properties, such as scatterer concentration and arrangement, for further applications in tissue characterization [28]. The backscattered envelope PDF tends to conform to a pre-Rayleigh distribution as the scatterers in the resolution cell have randomly-varying and high variation scattering cross-sections. When the resolution cell contains a large number of randomly distributed scatterers, the corresponding statistical envelope tends to approach to Rayleigh distribution. As the resolution cell contains periodically-located scatterers (or a high concentration of scatterers presented locally) in addition to randomly-distributed scatterers, the associated statistical distribution of the backscattered envelope tends to follow post-Rayleigh distribution [28]. To date, several statistical models, including Rayleigh, Rician, K, homodyned K, generalized K, Nakagami, and compound Nakagami distributions, have been utilized for characterizing tissue properties [29,30,31,32,33,34,35,36]. Aside from the Nakagami distribution, most of these mentioned models suffer either from short capability to describe all the features of signals received from different tissues or from a high computational complexity to estimate parameters, and that certainly tends to limit their practical applications in clinical diagnosis [37]. On the other hand, the Nakagami distribution is a general model capable of covering all the scattering conditions, including pre-Rayleigh, Rayleigh and post-Rayleigh distributions, encountered in medical ultrasound [28,34]. Specifically, the Nakagami parameter (*m*), which is a shape parameter and quantitative value for determining the statistics of an ultrasonic backscattered envelope, is capable of differentiating different scatterer concentrations in a medium. Furthermore, the Nakagami parameter has been applied to quantify scattering properties in various tissues, including bone, skin, breast, and blood [38,39,40,41].

As mentioned above, most of the previous studies employed ultrasound images to detect the morphology and echogenicity of the A1 pulley and flexor tendon to assess trigger finger. In addition to a lack of sufficient image resolution to directly distinguish the pulley tissue from the surrounding tissues, these previous studies also did not thoroughly characterize these tissues using quantitative ultrasonic parameters and statistical models of the ultrasonic backscattered signals. In the present study, in vitro measurements of the A1 pulley and surrounding tissues, including the hypodermis and superficial digital flexor tendon (SDFT), were performed employing a 30 MHz high-frequency ultrasound imaging system. High resolution ultrasound B-mode and Nakagami images were utilized to directly visualize variations of the A1 pulley and surrounding tissues. Subsequently, ultrasonic parameters, including *IB*, Nakagami parameter, sound speed, and attenuation coefficient, were further estimated to better characterize the A1 pulley and its surrounding tissues quantitatively.

## 2. Materials and Methods

### 2.1. Experimental Arrangement

The normal A1 pulley, hypodermis, and SDFT dissected from fresh cadaveric hands (14 samples of each tissue type) were prepared for in vitro experiments. The thicknesses of A1 pulley, hypodermis, and SDFT were 0.64 ± 0.14 mm, 2.60 ± 0.30 mm, and 1.84 ± 0.32 mm, respectively. The tissues to be interrogated were firstly fixed on a custom-made acrylic plate and then placed in saline solution. To avoid the tissues from floating during ultrasound scanning, the hypodermis was covered with a Tegaderm™ transparent film dressing (1624W, 3M, St. Paul, MN, USA). Subsequently, the compressional sound speed, backscattering and attenuation properties of the tissues were measured using a 30 MHz ultrasound transducer (NIH Ultrasonic Transducer Resource Center, USC, University Park, LA, USA). The distance between the transducer and tissue was adjusted to be approximately 7.7 mm to ensure the signal acquisition ocurred within the transducer’s focus zone. Measurements of the attenuation coefficient and sound speed of tissues were carried out using the broadband transmission substitution method [42], in which the distance between the transducer and plexiglass reflector was also related to the transducer’s focal length. To better explore the ultrasonic properties of A1 pulleys and surrounding tissues for the assessment of trigger finger, the B-mode images and measurements were performed from the sagittal and transverse planes of the finger. The sagittal plane corresponds to the scanning direction where the ultrasound probe is respectively across and along the fiber axis of the A1 pulley and the SDFT, whereas the transverse plane corresponds to the scanning direction along and across the fiber axis of the A1 pulley and SDFT, respectively. To align the scanning direction, the fiber direction of each A1 pulley and SDFT sample was carefully determined by observation. The adjustment of scanning direction from the transverse plane to the sagittal plane was achieved by rotating the tissues using a motorized rotation stage. For each sample, measurements were performed 10 times per each scan direction from different tissue locations. All of the experiments were performed in an air-conditioned laboratory at room temperature (24 °C to 25°C).

**Figure 1:**
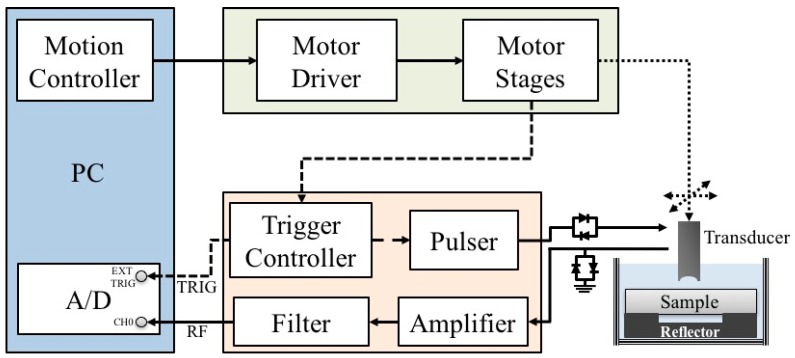
Block diagram of the experimental arrangement.

A schematic diagram detailing the arrangement of the high-frequency ultrasound system, which is similar to the arrangement in our previous studies [43,44,45], is shown in Figure 1. A 30 MHz single-element ultrasound transducer was employed for the generation and reception of ultrasound waves. The characteristics of the transducer are summarized in Table 1.

The transducer was driven by a pulser (Model 5900PR, Panametrics, Waltham, MA, USA) that is coupled with an expander (Matec Instruments Company, Northborough, MA, USA) to eliminate electrical noise. The received ultrasonic signals were connected to an electronic limiter (Matec Instruments Company) to protect the following low-noise amplifier (Model LN1000A, Amplifier Research, Souderton, PA, USA). Subsequently, the radio frequency ultrasonic signals were filtered by a bandpass filter (Model BIF-30, Mini-Circuits, Brooklyn, NY, USA) and then digitized by an 8-bit analog-to-digital converter (PXI 5152, National Instruments, Austin, TX, USA) with a 500 MHz sampling frequency. The transducer was mounted on the piezo-ceramic motor (HR8, Nanomotion Ltd., Yokneam, Israel) for sweeping the scanning at different locations of each sample. In addition, the transducer may also be flexibly moved to various directions controlled by the two axes of the stepping motors (CM1-C-17L30A, Cool Muscle, Osaka, Japan) which were controlled by a motion controller (DMC-1842, Galil Motion Control Inc., Rocklin, CA, USA). The computer program for data acquisition and motor control was developed using the LabVIEW software (National Instruments). The resolution of the B-mode image was measured from an 18 μm tungsten wire, where the −6 dB axial and lateral resolution were measured approximately to be 33 and 72 μm, respectively. Each acquired B-mode tissue image covered an area of 5 mm (width) × 1.5 mm (depth) from 250 A-lines at 20 μm intervals.

Following the ultrasonic measurements, the slices of A1 pulley, hypodermis, and SDFT were prepared by a typical processing sequence, including sample fixations in a 10% neutral-buffered formalin, dehydration with ethanol, clean up with xylene, infiltration with paraffin, and then embedding in paraffin. Finally, each slice of tissue sample was cut into 5 μm thickness and then stained with hematoxylin and eosin (H&E). The histological section of tissue samples was observed with an optical microscope (ECLIPSE TS100-F, Olympus Corporation, Center Valley, PA, USA), and the corresponding images were captured with a digital charge-coupled device (Evolution MP, Media Cybernetics Inc., Rockville, MD, USA).

### 2.2. Ultrasonic Parameters Analysis

The sound speed of tissues (*c_m_*) was measured using the following equation [46]:

(1) $$c_{m}=\left(\frac{T_{w}-T_{m}}{t_{2}-t_{1}}+1\right)c_{w},$$

where *T_m_* and *T_w_* respectively denote the time-of-flight of the propagation path with and without the tissue sample in it; *t*_1_ and *t*_2_ are respectively the time-of-flight for the incident pulse traveling from the transducer to the front and rear interface of the tissue sample, and *c_w_* represents the sound speed of saline solution which is around 1504.1 ± 0.97 m/s. The sound speed not only corresponds to tissue properties, but also may be applied to estimate tissue thickness for attenuation measurement and signal compensation.

The attenuation coefficient of a tissue (*α*(*f*), dB/mm) as a function of frequency within the transducer frequency response bandwidth may be estimated using the following equation [42]:

(2) $$\alpha (f)=\frac{10}{2d}\left(\log\frac{S_{w}(f)}{S_{m}(f)}\right),$$

in which *d* is the thickness of the tissue sample, and *S_w_*(*f*) and *S_m_*(*f*) denote the power spectra of reflecting echoes received from the reflector in the saline solution and reflector with the tissue sample interposed, respectively. The frequency-dependent attenuation coefficient was estimated within a frequency range from 22 to 36 MHz corresponding to a −6 dB bandwidth of the transducer, and then the attenuation slope, in units of dB/mm-MHz, was calculated by linear regression. The estimated attenuation was applied to compensate losses of echo signals, in which the function for attenuation compensation, *A*(*f*), is given [47] as follows:

(3) $$A(f)=e^{4\alpha_{S}(f)d_{S}}e^{4\alpha_{T}(f)d_{T}}{\left[\frac{2\alpha_{T}(f)L}{1-e^{2\alpha_{T}(f)L}}\right]}^{2}{\left[1+{\left(\frac{\alpha_{T}(f)L}{\pi }\right)}^{2}\right]}^{2},$$

where *d_S_* denotes the distance between the transducer and the tissue surface; *d_T_* is the distance between the tissue surface and the region of interest (ROI); *L* represents the length of ROI in mm; *α_S_*(*f*) and *α_T_*(*f*) are the frequency-dependent attenuation coefficients of the saline solution and the tissue sample, respectively, in units of Np/mm. The frequency-dependent attenuation slope of the saline solution was estimated to be 2.96 × 10^−5^ Np/mm/MHz^2^.

Ultrasonic backscattering signals are known to correspond to the microstructure and scattering properties of biological tissues. The *IB*, frequently adopted to quantify the strengths of ultrasonic backscatter signals, is defined as the frequency average of the backscattering transfer function of a sample volume over the transducer’s bandwidth normalized relative to that of from a flat reflector. The *IB* can be measured by the following equations [48]:

(4) $$IB=\frac{1}{f_{2}-f_{1}}\int_{f_{1}}^{f_{2}}\frac{\left|S_{ROI}(f)\right|}{\left|S_{ref}(f)\right|}df\;,$$

(5) $$S_{ROI}(f)=\frac{A(f)}{N}\sum_{i=1}^{N}S_{i}(f),\;i=0,\;1,\;2,\;\ldots ,\;(N-1),$$

where *f*_1_ and *f*_2_ respectively denote the lower and upper frequencies within the −6 dB bandwidth of the ultrasonic spectrum, and *S_ref_*(*f*) and *S_ROI_*(*f*) represent respectively the power spectra of echoes received from a stainless steel reflector and that of backscattered signals obtained from the tissue. The *S_ROI_*(*f*) was computed by averaging the power spectrum, *S_i_*(*f*), from *i* = 0, 1, 2, …, (*N* – 1) A-lines of attenuation-compensated signals within the ROI using fast Fourier transform with a Hanning window.

The PDF of ultrasonic backscattered strengths is known to correspond to the concentration and arrangement of scatterers, in which Nakagami distribution has been verified to be a general statistical model with relatively less computational complications able to characterize biological tissues [28,34]. The PDF of Nakagami distribution, *f*(*R*), of the ultrasonic backscattered envelope (*R*) is given by [34]:

(6) $$f(R)=\frac{2m^{m}R^{2m-1}}{\Gamma (m)\Omega^{m}}\cdot e^{\left(-\frac{m}{\Omega }R^{2}\right)}\cdot U(R),$$

where Γ(·) and *U*(·) represent the gamma function and unit step function, respectively. Both the scaling parameter and the Nakagami parameter (*m*) governing the characteristics of the Nakagami distribution are formulated as:

(7) $$\Omega =E(R^{2}),$$

and:

(8) $$m=\frac{{\left[E(R^{2})\right]}^{2}}{E{\left[R^{2}-E(R^{2})\right]}^{2}},$$

where *E*(·) is the statistical mean. The scaling parameter readily corresponds to the average power of the backscattered envelope. The *m* is the shape parameter of Nakagami distribution and is associated with the distribution of backscattered signals in the resolution cell. The backscattered envelope tends to conform to a pre-Rayleigh distribution, with *m* smaller than 1. The statistical envelope approaches the Rayleigh distribution, with *m* equal to 1. The statistical distribution of the backscattered envelope tends to be post-Rayleigh distributed, with *m* larger than 1. Nakagami imaging for exploring local variation of signals was further implemented by a stable approach using a square sliding window that composed of three pulse lengths [37]. The pseudo-color for Nakagami image displaying was designated corresponding to the magnitude of each *m* ranging from 0 to 2.

In the present study, the ultrasonic backscattering signals for calculating the *IB* and *m* parameter were acquired from a ROI of 1 mm (width) × 0.4 mm (depth) covering the focal region of the transducer. The data analysis and imaging formation were implemented using the MATLAB software (The MathWorks, Natick, MA, USA). Analysis of variance was applied to investigate the significance of sound speed, attenuation slope, attenuation coefficient, *IB* and *m* on the differentiation between the A1 pulley and surrounding tissues. In addition, paired *t*-test was used to study the significance of *IB* and *m* on the differentiation between the quantitative parameters estimated from images acquired from the transverse and sagittal planes of the samples. A *p*-value smaller than 0.05 was regarded as significant. The statistical analyses were conducted using SigmaPlot software (Systat Software Inc., San Jose, CA, USA).

## 3. Results

**Figure 2:**
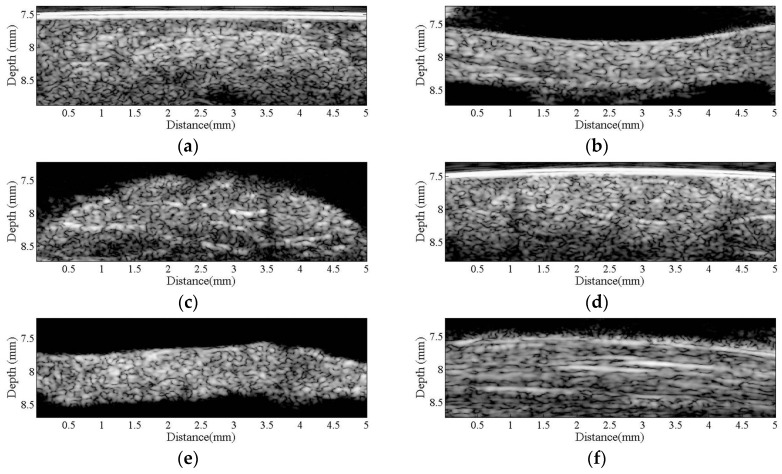
B-mode images of (**a**) hypodermis; (**b**) A1 pulley; and (**c**) SDFT were acquired in the sagittal plane, and those acquired in the transverse plane correspond to (**d**–**f**), respectively.

Figure 2 is a series of B-mode images of (a) hypodermis; (b) A1 pulley; and (c) SDFT acquired in the sagittal plane, and those acquired in the transverse plane corresponding to (d) to (f), respectively. The textures of these B-mode images in terms of granular size and orientation provide a much better resolution than those of clinical ultrasound images, which were implemented using lower frequency ultrasound, to differentiate the varied structures of tissues. However, the texture patterns of the hypodermis region are not significantly different between images acquired from the transverse and sagittal planes. The brightness echo in the B-mode images of the hypodermis of about 7.5 mm corresponds to the Tegaderm^TM^ transparent dressing. Specifically, the textures of A1 pulley and SDFT B-mode images acquired from the transverse direction differed from those of the sagittal direction. More regulatory patterns were found in the A1 pulley images acquired in the transverse plane than that those in the sagittal plane. The images of SDFT in the sagittal plane have apparent fibrillar patterns. This texture discrepancy certainly corresponds to the histological structure and composition of the tissue.

**Figure 3:**
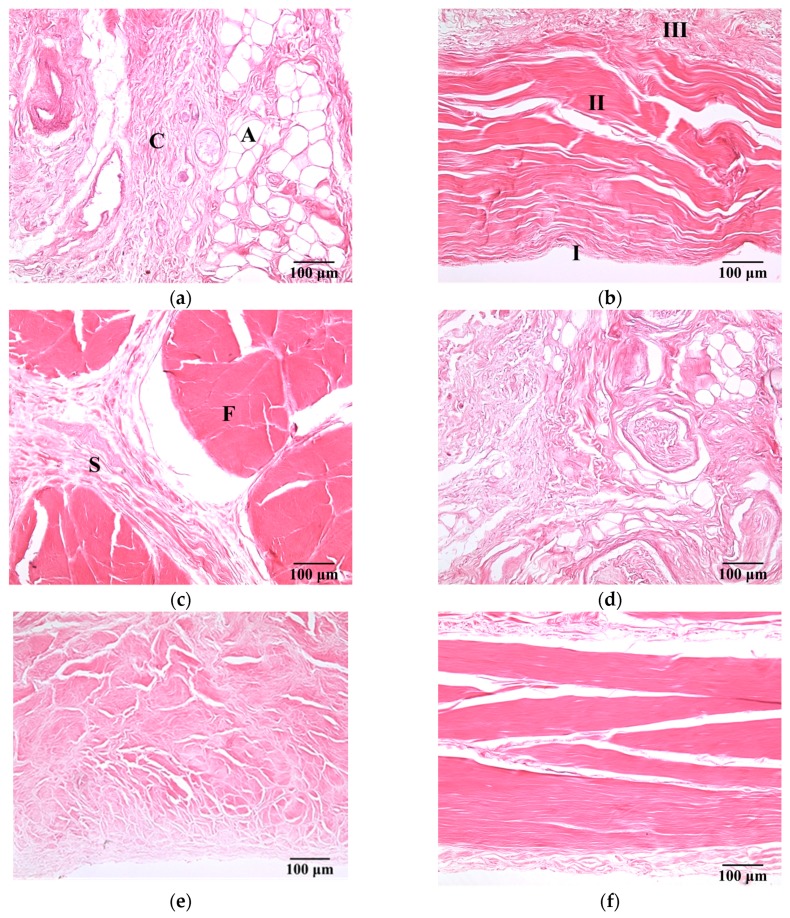
H&E-stained sections of (**a**) hypodermis; (**b**) A1 pulley; and (**c**) SDFT were sliced in the sagittal plane, and those sliced in the transverse plane corresponded to (**d**) to (**f**). (100× magnification). The symbols C and A indicate the connective tissue and adipose lobules in the hypodermis, respectively. The pulley is made up of three layers: I = innermost layer, II = middle layer, III = outermost layer. The symbols F and S indicate the connective tissue sheath and fascicle in the SDFT, respectively.

Figure 3 present a series of microscopic histological sections corresponding to local structures of the hypodermis, A1 pulley, and SDFT in the sagittal and transverse planes. The adipose lobules and connective tissues of the hypodermis in the sagittal plane, as shown in Figure 3a, tended to distribute similarly to those of in the transverse plane, as shown in Figure 3d. The A1 pulley in the sagittal plane, as seen in Figure 3b, clearly shows that it was composed of a three-layer structure in which the outermost layer, close to the hypodermis corresponded to areolar tissue. The middle layer was mostly composed of dense collagen bundles oriented perpendicular to the long axis of the digit; the innermost layer, neighbored to the SDFT, consisted of collagen bundles oriented in parallel to the long axis of the digit to form the gliding surface. The SDFT was formed by binding the groups of fascicles and encasing in connective tissue sheaths, as shown in Figure 3c. The fascicle was composed of dense collagen fibrils oriented parallel to the long axis of the digit, as shown in Figure 3f. The striking orientation of collagen bundles in the A1 pulley and SDFT is the primary tissue responsible for the fibrillar texture patterns seen in the B-mode images.

The compressional sound speed, the attenuation coefficient and *IB* of the hypodermis, A1 pulley, and SDFT, were calculated to further quantitatively characterize the tissue samples. The compressional sound speeda of the hypodermis, A1 pulley, and SDFT were measured as 1492.3 ± 12.7, 1624.4 ± 24.1, and 1633.2 ± 15.2 m/s, respectively. The compressional sound speed of the hypodermis apparently was thus much smaller than those of the A1 pulley and SDFT (*p* < 0.001). The sound speeds of the A1 pulley and SDFT were not significantly different (*p* = 0.308).

**Figure 4:**
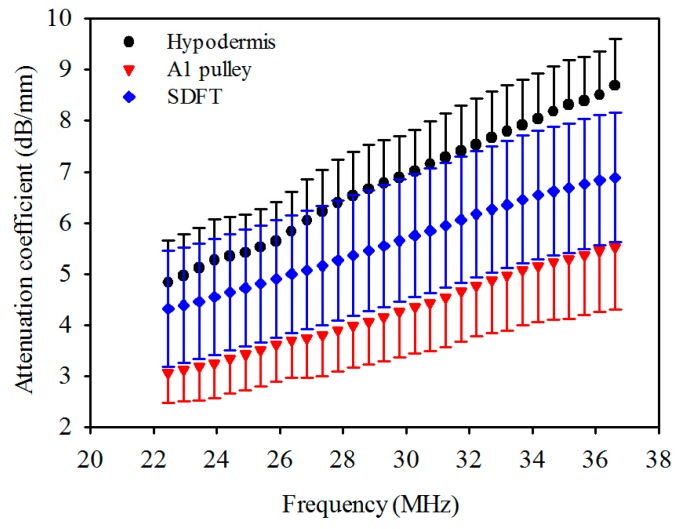
Attenuation coefficient as a function of frequency of hypodermis, A1 pulley, and SDFT. The error bars represent the standard deviation.

The attenuation coefficients of the hypodermis, A1 pulley, and SDFT as a function of frequency can be found in Figure 4. The linear regression fitting of the attenuation slope of the hypodermis, A1 pulley, and SDFT were 0.274 ± 0.042, 0.183 ± 0.050, and 0.190 ± 0.027 dB/mm/MHz, respectively, with a R-square value larger than 0.9. The attenuation slope of hypodermis was significantly larger than those of A1 pulley and SDFT (*p* < 0.001), and that of A1 pulley had a similar tendency to SDFT (*p* = 0.891). The attenuation coefficients, corresponding to the use of 30 MHz ultrasound, of hypodermis, A1 pulley, and SDFT were estimated to be 7.0 ± 0.81, 4.3 ± 0.90, 5.8 ± 1.2 dB/mm, respectively. The attenuation coefficient tends to differ significantly between the A1 pulley and hypodermis (*p* < 0.001), and that of the A1 pulley and SDFT is not much different (*p* = 0.891). Moreover, the results of compressional sound speed and attenuation coefficient were applied to compensate the attenuation of tissues for the estimation of *IB*.

**Figure 5:**
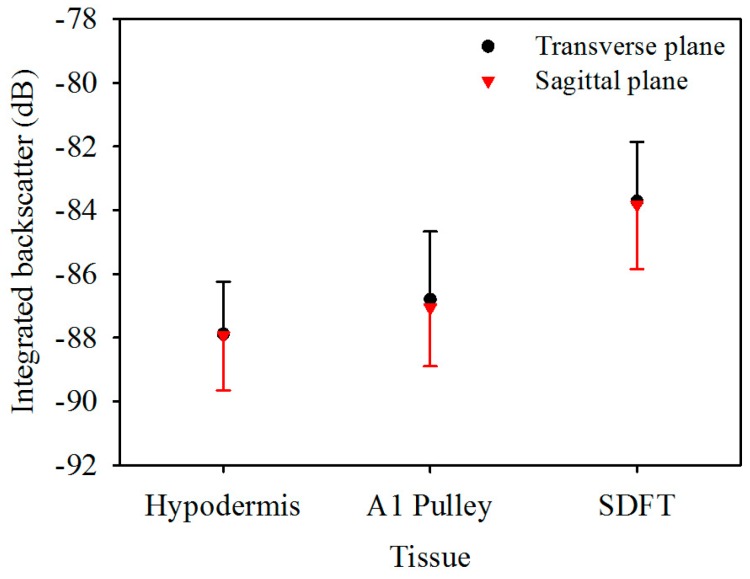
Integrated backscatter of hypodermis, A1 pulley, and SDFT. The error bars represent the standard deviation.

Figure 5 is the *IB* of ultrasonic backscattering signals of the hypodermis, A1 pulley, and SDFT in the transverse plane, calculated respectively to be −87.9 ± 1.6, −86.8 ± 2.1, and −83.7 ± 1.9 dB, and those in the corresponding sagittal plane were calculated to be −87.9 ± 1.7, −87.1 ± 1.8, and −83.8 ± 2.0 dB. These results indicated that the estimated *IB* of the hypodermis, A1 pulley, and SDFT in the transverse plane were similarly to those in the sagittal plane. According to these statistical analysis results, the *IB* differed significantly between the signals of the A1 pulley and SDFT, regardless of whether the signals were acquired from the transverse or sagittal planes of the tissue. Nevertheless, the *IB* of the A1 pulley is not much different from that of the hypodermis.

**Figure 6:**
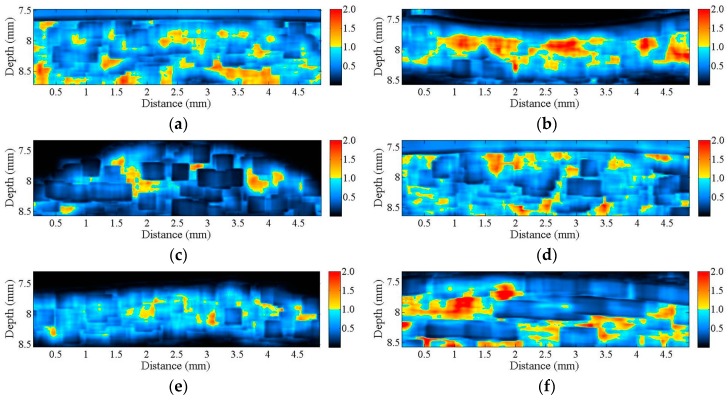
Nakagami images corresponding to the B-mode images in Figure 2 of (**a**) hypodermis; (**b**) A1 pulley; and (**c**) SDFT in the sagittal plane; and of (**d**) hypodermis; (**e**) A1 pulley; and (**f**) SDFT in the transverse plane.

**Figure 7:**
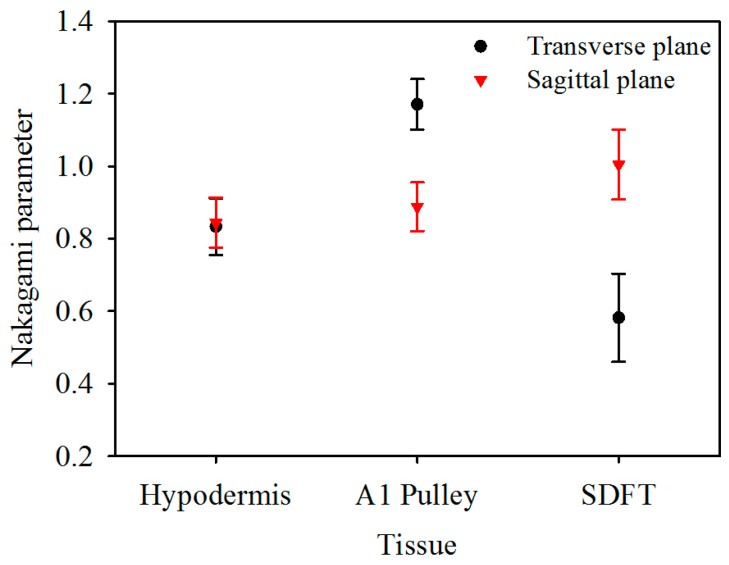
Nakagami parameter of hypodermis, A1 pulley, and SDFT. The error bars represent the standard deviation.

The corresponding Nakagami image results, shown in Figure 6, can be readily correlated to the B-mode images in Figure 2. A large difference in the distribution of *m* parameters may be found from the Nakagami images of both the corresponding A1 pulley and SDFT images in the transverse and sagittal planes, whereas not much difference was found between the Nakagami images of the hypodermis acquired in the transverse and sagittal planes. Furthermore, the average *m* values from the ROI of the hypodermis, A1 pulley, and SDFT in the transverse plane were estimated to be 0.83 ± 0.08, 1.17 ± 0.07, and 0.58 ± 0.12, respectively; while those of in the sagittal plane were 0.85 ± 0.07, 0.89 ± 0.07, and 1.00 ± 0.10, respectively, as shown in Figure 7.

The *m* of the A1 pulley, estimated from signals of the transverse plane, was significantly larger than those of both the hypodermis (*p* < 0.001) and SDFT (*p* < 0.001). For the signals acquired from sagittal plane, the *m* tends to differ significantly between the A1 pulley and SDFT (*p* < 0.001), and that of the A1 pulley and hypodermis are not much different (*p* = 0.299). A significant difference can be readily discerned *m* parameters of the A1 pulley (*p* < 0.001) and SDFT (*p* < 0.001) estimated between the signals acquired from the transverse and sagittal planes. Whereas the *m* of hypodermis estimated from signals acquired from the transverse plane did not differ significantly from that of the sagittal plane (*p* = 0.427). The statistical analyses of both *IB* and *m* parameters are summarized in Table 2.

## 4. Discussion

**Figure 8:**
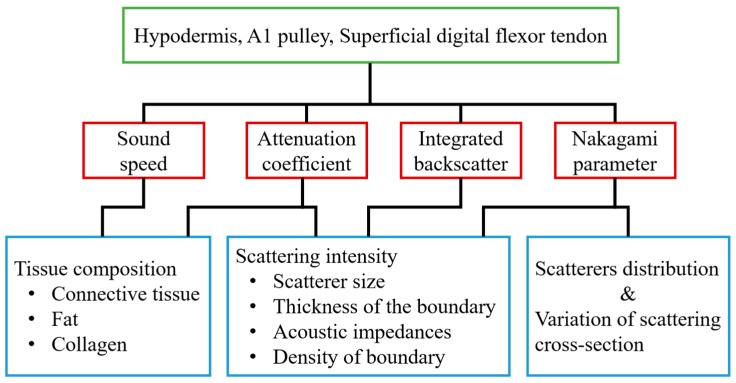
The major factors associated with the quantitative parameters used in this study.

With 30 MHz high-frequency B-mode images, the corresponding tissue structures may be readily differentiated, and the gray scale and quality of these high-frequency images were found to be easily affected by several system settings, such as system gain, time-gain compensation, dynamic range of image, and other procedures related to the signal and image processing scheme used. On the other hand, several acoustic parameters, including sound speed, attenuation coefficient, *IB*, and *m*, have been shown capable of quantifying the composition and structure of tissues. In the present study, these parameters were measured for characterizing the normal hypodermis, A1 pulley, and SDFT in vitro. The sound speed, attenuation coefficient, *IB*, and *m* for characterizing the normal A1 pulley and adjacent tissues are discussed in sequence in the following paragraphs. The major factors related to the quantitative parameters used in this study are illustrated in Figure 8.

The sound speed of the hypodermis measured as 1492.3 m/s in this study was faster than that of 1465 m/s reported by Errabolu et al. [49]. This discrepancy could be associated with the variations of the components in the hypodermis sample. The hypodermis appears as thin and sparse collagen fibers that mostly consist of fat held together with connective tissues. The composition ratio between fat and connective tissue, size of fat, and the density of connective tissue in the hypodermis vary among body sites. The structures of the hypodermis of the palm, like the slices shown in Figure 3a,d, contain a large number of dense connective tissues and white fibers which bind the skin firmly to the deep tissues [50]. Previous research has indicated that the sound speed of tissues tends to decrease in tissue containing a large predominance of fat and increase in that which contains a large number of collagen fibers [51]. Therefore, the increase of sound speed of the hypodermis could correspond largely to the amount of connective tissue in the hypodermis. Moreover, due to the fact they mainly consist of densely arranged collagen fibers predominantly composed of type I collagen, the sound speed of both the A1 pulley and SDFT were measured to be significantly faster than that of the hypodermis. The sound speed of the tendon was found to vary among different regions and animals primarily because of the variation of collagen content [52]. For example, the sound speed of equine SDFT and deep digital flexor tendon (DDFT) corresponding to the propagation of ultrasound across the fiber axis of the tendon were measured to be 1604 and 1650 m/s at 0 °C, respectively [53]; that of the equine SDFT was measured to be 1668 m/s at 20 °C [53]; that of the bovine DDFT was measured to be 1601 m/s at room temperature [54]. Furthermore, with the measured sound speed of 1624.4 m/s, the average thickness of A1 pulley (0.65 ± 0.11 mm) agrees well with that measured from histological slides (0.64 ± 0.14 mm), and that was slightly larger than reported, ranging from 0.38 to 0.6 mm [12,14,15]. The measured sound speed of the A1 pulley was larger than 1540 m/s, which is commonly implemented in commercial ultrasound scanners, and that leads the estimation of the A1 pulley thickness to be only 5.2% (0.031 mm difference) in comparison with the actual thickness of 0.6 mm. Despite of factors of variations caused by sample differences and also sound speeds, the lower resolution of the images associated with ultrasound frequencies lower than 20 MHz certainly will affect the manual boundary estimation of A1 pulley in clinics and also will increase the variations in thickness measurement [55].

The results of in vivo experiments indicated that the median attenuation coefficient slope and attenuation coefficient of the hypodermis in the human forearm were measured to be 0.274 dB/mm/MHz and about 5.8 dB/mm at 30 MHz, respectively [56]. These results were smaller than the attenuation of the palmar hypodermis just measured in the present study, a 0.279 dB/mm/MHz median attenuation coefficient slope and a 7.0 dB/mm of attenuation coefficient at 30 MHz. Due to the fact that the attenuation of fat tissue decreases as the temperature increases and the fact that the content of collagen fibers is positively related to tissue attenuation [51,57], these differences in attenuation may be related to the variations in temperature and the composition of the hypodermis. In addition, although highly-aligned collagen fibers are a major part of the composition of the A1 pulley and SDFT, this study indicated the attenuation coefficient slope to be similar between that of the A1 pulley and SDFT, whereas the attenuation coefficient of the SDFT was significantly larger than that of the A1 pulley (Figure 4). Similar attenuation tendencies were found in equine SDFT and DDFT as reported by Miles [53]. Because frequency-dependent attenuation coefficients experience energy losses due to both absorption and scattering in the tissues, the difference in attenuation coefficient between the A1 pulley and SDFT would be caused by the effect of scattering in the tissues.

Previous studies have demonstrated that the intensity of backscattering signals from a tendon had high anisotropy due to the high alignment of collagen fibers in fixed tendons. In other words, when the ultrasonic propagation was perpendicular to the fiber axis of the tendon, the intensity of the backscattering signals was maximal, and the minimum intensity of the backscattering signals occurred for propagation that was parallel to the fiber axis [27]. Although the fixation by formalin preserved the backscattering coefficients of tendon to a reasonable extent, the acoustic scattering strength of tendon would be varied with the angle of ultrasonic insonification whether the tendon was fixed by formalin or not [27]. Because the A1 pulley also consists of aligned collagen fibers, the A1 pulley would have this backscattering anisotropy. In this study, regardless of whether the tissues were imaged in the transverse plane or in the sagittal plane, the ultrasonic insonification was perpendicular to the fiber axis of both the A1 pulley and SDFT. Therefore, the *IB* of the A1 pulley and SDFT acquired on the transverse plane tended to be similar to those acquired in the sagittal plane, as shown in Figure 5. Moreover, the *IB* of the hypodermis of both acquired plane were consistent due to the distributions of fat and connective tissue in the hypodermis are irregular. The intensity of backscatter signals from A1 pulley and SDFT would therefore depend on the thickness of the boundaries, the contrast of acoustic impedances at boundaries, and the density of boundaries associated with the size of the collagen bundles/fibrils/fascicles [58]. The thickness of the inter-collagen-bundle space were measured using H&E-stained sections. The thickness of A1 pulley is ranged between 0.1 and 10 μm. The thickness of the inter-collagen-bundle space in the SDFT, which contains interfascicular space and interfibrillar space, ranged between 1 and 80 μm. If the thickness of the inter-collagen-bundle space is larger than the wavelength (approximately 54 μm at 30 MHz), strong echoes are acquired from the space, and no specific structures can be observed for the thickness of an inter-collagen-bundle smaller than the wavelength [59]. Hence, a greater thickness in the inter-collagen-bundle space of the SDFT compared with the A1 pulley would lead the SDFT to have higher backscattering intensity.

**Figure 9:**
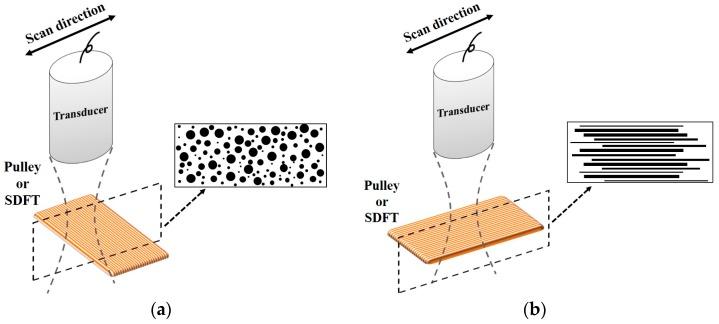
Diagrams of the scatterer distribution in the A1 pulley and SDFT as the ultrasonic transducer imaged (**a**) across the fiber axis and (**b**) along the fiber axis.

Besides quantifying the tissue properties by *IB*, the *m* estimated from the PDF of the ultrasonic backscattered envelopes and the Nakagami image implemented from the envelopes of a local sliding window tended to be affected minimally by the system settings. As shown in Figure 6, both *m* values of the hypodermis acquired from the transverse and sagittal planes are similar because the distributions of fat and connective tissue in the hypodermis do not have particular orientations. The *m* of the hypodermis is smaller than 1, indicating that the PDF of the corresponding backscatter envelope is pre-Rayleigh distributed. From a histological viewpoint, the hypodermis is mostly composed of fat with a diameter in a range from 10 to 80 μm and connective tissue which has a size ranging from tens of microns to tens of angstroms. Therefore, the effective scatterers in each resolution cell of the incident ultrasound tends to display high variations, and that leads the corresponding PDF of backscatter envelopes to be pre-Rayleigh distributed. Furthermore, when the scan direction of the ultrasound was along the fiber axis of the collagen fiber, indicating that the A1 pulley and SDFT was imaged in the transverse plane and sagittal plane, respectively, the *m* values were distinctly larger than those acquired in the scan direction across the fiber axis. Due to the fact that the backscattered signals were mainly dominated by scattering from the interfaces between collagen bundles, the concentration and arrangement of the scatterers would be regarded as the distributions of inter-collagen-bundle spaces. When the scan direction was perpendicular to the fiber axis of the A1 pulley and SDFT, due to the fact most scatterers may be regarded as having randomly varying scattering cross-sections with high variations as illustrated in Figure 9a, the PDF of the backscattered envelope tended toward a pre-Rayleigh distribution associated with a *m* smaller than 1. Specially, because the degree of scatterer variance in the SDFT was larger than that in the A1 pulley, the *m* of the SDFT was smaller than that of the A1 pulley. When the scan direction was along the fiber axis of the A1 pulley and SDFT, because the resolution could contain periodically-located scatterers with high variations of scattering cross-sections (illustrated in Figure 9b), the PDFs of the backscattered envelopes simultaneously had both pre-Rayleigh and post-Rayleigh distributions, as shown in Figure 6b,f. The SDFT having a higher degree of scatterer variance than the A1 pulley caused the *m* of the SDFT to be smaller than that of the A1 pulley.

These results indicated that *m* parameter seems to be more robust to quantitatively distinguish properties of the A1 pulley from adjacent tissues for the scanning direction in the transverse plane. Due to the fact our tissue samples were dissected from fresh cadaveric hands, the density and compressibility of the tissues from a cadaver could be different from that of a live patient. Further clinical studies to explore this issue are warranted. According to results in this study, the B-mode image is better acquired in the transverse planes when high-frequency ultrasound is applied to measure the A1 pulley in vivo. The results also suggest the measurement method in conjunction with *m* parameter to be applied to quantitatively differentiate the A1 pulley. In addition, the assessment of the diseased A1 pulley in trigger finger using high-frequency ultrasound and ultrasonic parameters could be of interest to be studied in the future.

## 5. Conclusions

A high frequency ultrasound system with a 30 MHz central frequency was used to apply to measure ultrasonic backscattered signals reflected from the A1 pulley, hypodermis, and SDFT in both transverse and sagittal planes. High-frequency ultrasound images have been shown to provide a sufficient resolution to differentiate variations among each type of tissue structure. Specifically, the textures of transverse B-mode images, which were acquired from the A1 pulley and SDFT, differed significantly from those of sagittal B-mode images. In addition to acquiring B-mode images, ultrasonic parameters, including compressional sound speed, the attenuation coefficient, *IB* and *m*, were calculated from tissue samples to characterize the tissue properties. The compressional sound speed of the hypodermis, A1 pulley, and SDFT corresponded to 1492.3 ± 12.7, 1624.4 ± 24.1, and 1633.2 ± 15.2 m/s, respectively. The attenuation slope of the hypodermis (0.274 ± 0.042 dB/mm/MHz) was shown to be significantly larger than that of the A1 pulley (0.183 ± 0.050 dB/mm/MHz) and SDFT (0.190 ± 0.027 dB/mm/MHz). Although the attenuation coefficient slope of the A1 pulley was similar to that of the SDFT, the attenuation coefficient of the SDFT was significantly larger than that of the A1 pulley. When the scan direction was in the transverse plane, the *m* could distinguish the A1 pulley (1.17 ± 0.07) from its surrounding tissues (0.83 ± 0.08 of hypodermis and 0.58 ± 0.12 of SDFT). While the A1 pulley was imaged in the sagittal plane, the *m* of the A1 pulley (0.89 ± 0.07) was similar to that of the hypodermis (0.85 ± 0.07). The *IB* differed significantly between the A1 pulley (−87.1 ± 1.8 dB) and SDFT (−83.8 ± 2.0 dB) acquired in the sagittal plane, but the A1 pulley and hypodermis exhibited similar *IB*. This study demonstrated that high-frequency ultrasound images in conjunction with ultrasonic parameters are able to characterize the A1 pulley and surrounding tissues well for further diagnosis of trigger finger syndrome.

## Figures and Tables

**Table 1:** Characteristics of the applied transducer.

Central Frequency	30 MHz
−6 dB bandwidth	14 MHz
*f-*number	1.6
Focal length	8 mm
Aperture size	5 mm
Axial resolution	35 μm
Lateral resolution	72 μm

**Table 2:** The *p* value of the integrated backscatter and Nakagami parameter with different tissues acquired in the transverse and sagittal planes. The subscript of *t* and *s* indicate direction of images acquired from the transverse plane and sagittal plane, respectively.

Tissue		Parameter	
		Integrated Backscatter (dB)	Nakagami Parameter
Hypodermis*_t_*	Hypodermis*_s_*	0.807	0.427
A1 pulley*_t_*	A1 pulley*_s_*	0.076	<0.001
SDFT*_t_*	SDFT*_s_*	0.5	<0.001
Hypodermis*_t_*	A1 pulley*_t_*	0.227	<0.001
Hypodermis*_t_*	SDFT*_s_*	<0.001	<0.001
A1 pulley*_t_*	SDFT*_s_*	<0.001	<0.001
Hypodermis*_s_*	A1 pulley*_s_*	0.407	0.299
Hypodermis*_s_*	SDFT*_t_*	<0.001	<0.001
A1 pulley*_s_*	SDFT*_t_*	<0.001	<0.001
